# A Network Pharmacology Approach to Explore Mechanism of Action of Longzuan Tongbi Formula on Rheumatoid Arthritis

**DOI:** 10.1155/2019/5191362

**Published:** 2019-01-17

**Authors:** An Huang, Gang Fang, Yuzhou Pang, Zongran Pang

**Affiliations:** ^1^Guangxi University of Chinese Medicine, Nanning, China; ^2^College of Pharmacy, Minzu University of China, Beijing, China

## Abstract

Longzuan Tongbi Formula (LZTB) is an effective proved prescription in Zhuang medicine for treating active rheumatoid arthritis (RA). However, its active ingredients, underlying targets, and pharmacological mechanism are still not clear in treating RA. We have applied network pharmacology to study LZTB and found that 8 herbs in LZTB and 67 compounds in the 8 herbs are involved in the regulation of RA-related genes; we have conducted pathway analysis of overlapping genes and found that 7 herbs participate in the regulations of 24 pathways associated with RA and that 5 herbs in the 7 herbs and 25 compounds in the 5 herbs participate in the regulation of hsa05323 (rheumatoid arthritis). The results indicated that all herbs in LZTB and some compounds in those herbs participate in the treatment of RA; 25 compounds are main active ingredients and hsa05323 (rheumatoid arthritis) is the major pathway in the treatment of RA. We have also found that three pathways (inflammatory mediator regulation of TRP channels, PPAR signaling pathway, and mTOR signaling pathway) might have some effect on the treatment of RA.

## 1. Introduction

Rheumatoid arthritis (RA) is a chronic, systemic, and autoimmune disease. It can lead to irreversible joint destruction and deformity, seriously affecting people's quality of life [1]. Disease-modifying antirheumatic drugs, anti-inflammatory drugs, and painkillers have long been used to treat RA [2]. With the development of medicine, more and more attention is paid to diverse therapeutic methods, and a number of patients select adjuvant therapies and complementary and alternative medicine to treat their RA. As a critical component of complementary and alternative medicine, Zhuang medicine plays an important role in treating RA. Longzuan Tongbi Formula (LZTB) is an effective proven prescription in Zhuang medicine for treating active RA. It consists of* Toddalia asiatica* (TA),* Kadsura coccinea* (KC),* Alangium chinense* (AC),* Sinomenium acutum* (SA),* Bauhinia championii* (BC),* Spatholobus suberectus *(SS),* Zanthoxylum nitidum *(ZN), and* Ficus hirta Vahl* (FHV). Clinical studies have shown that LZTB can significantly reduce RA patients' erythrocyte sedimentation rate, and that it has a good therapeutic effect on improving RA patients' morning stiffness and joint pain [3]. However, its active ingredients, underlying targets, and pharmacological mechanism in treating RA are still not clear.

Compound preparations in traditional medicine can act on multiple targets through multicomponent reaction, play an indispensable role in core pathways in diseases, and help achieve the purpose of treating diseases [4]. Much attention has been given to the study of the multicomponent and multitarget action mechanism. With the rapid development of bioinformatics, network pharmacology has become a new way to effectively and systematically study the mechanism of action, safety, and other aspects of compound preparations in traditional medicine [5–7]. For example, Tang et al. have applied network pharmacology to study the mechanism of action of XuanHuSuo Powder in treating osteoarthritis [8], Liu et al. have adopted a network pharmacology approach in exploring the pharmacological mechanism of Xiaoyao Powder on anovulatory infertility [9], and Li et al. have analyzed the therapeutic effects of Zi Dian Fang on immune thrombocytopenic purpura through the integration of network pharmacology and metabolomics [10]. In network pharmacology, the relationship between drugs, their targets, and diseases can be studied through scientific calculations and displayed through visual networks [5, 6]. Therefore, a network pharmacology approach has been adopted in this study to explore the mechanism of action of LZTB in treating rheumatoid arthritis.

## 2. Material and Methods

### 2.1. Prediction of Targets of LZTB for RA

BATMAN-TCM platform (http://bionet.ncpsb.org/batman-tcm/) was used to predict targets of LZTB for RA. On this platform, drug similarity is used to predict underlying drug targets. Its core idea is to calculate drug similarity and rank potential drug-target interactions by comparing potential and known drug-target interactions [11]. On BATMAN-CTM platform, the full pinyin of all herbs in LZTB was input in the order of principal and auxiliary drugs together with* Ficus hirta Vahl* (FHV) as major compound information (PubChem_ID: the details are described in Table S01) [12]; cutoff parameter was set at 10, and targets of LZTB for RA were predicted.

### 2.2. Prediction of RA Targets

DisGeNET (http://www.disgenet.org) database was applied to acquire RA targets [13].

### 2.3. Network Construction

#### 2.3.1. RA Target Network

RA targets were acquired through DisGeNET, Gene_IDs of targets were input into String (http://string-db.org/, version10.5) [14], species were limited to “Homo sapiens”, and confidence score was set at 0.9. After PPI was acquired, it was imported into Cytoscape3.6.1 [15] to construct the network.

#### 2.3.2. LZTB Target Network

Targets of LZTB were acquired through BATMAN-TCM and text retrieval, and herb-compound target network was constructed through Cytoscape3.6.1.

#### 2.3.3. Compound Target-RA Target Network

Draw Venn Diagram (http://bioinformatics.psb.ugent.be/webtools/Venn/) was applied online to find the intersection of LZTB targets acquired through BATMAN-TCM and RA targets acquired through DisGeNET. Cytoscape3.6.1 was applied to construct LZTB-RA interaction network.

### 2.4. Cluster

In large protein-protein interaction (PPI) networks, topological modules or clusters are defined as the regions where molecular complexes are densely connected [16, 17], and they have the property of pure network. Functional modules refer to aggregation of nodes in the same network that have similar or related functions. Disease modules refer to multiple networks that are united to destroy cellular functions and lead to particular diseases [18]. As topological, functional, and disease modules have the same meaning in the network, functional modules and topological modules correspond, and diseases can be regarded as the results of disturbed and destroyed functional modules [17]. Clusters of each network were obtained by analyzing the corresponding network through MCODE, an add-in of Cytoscape [16].

### 2.5. Enrichment Analysis

#### 2.5.1. Gene Ontology (GO) Enrichment Analysis

In the field of biology, GO analysis is widely used to analyze the functions of genes [19]. It is mainly applied to describe the functions of gene products, including cell function, molecular function, and biological function. In this study, the ClusterProfiler package of R3.5.0 [20] was adopted to conduct GO enrichment analysis on overlapping targets. Based on communalities of annotations, cluster analysis was conducted through fuzzy clustering algorithms to cluster annotation terms and score clusters. Higher scores represent greater importance of represented genes in the lists of genes.

#### 2.5.2. KEGG Pathway Enrichment Analysis

**Figure 1:**
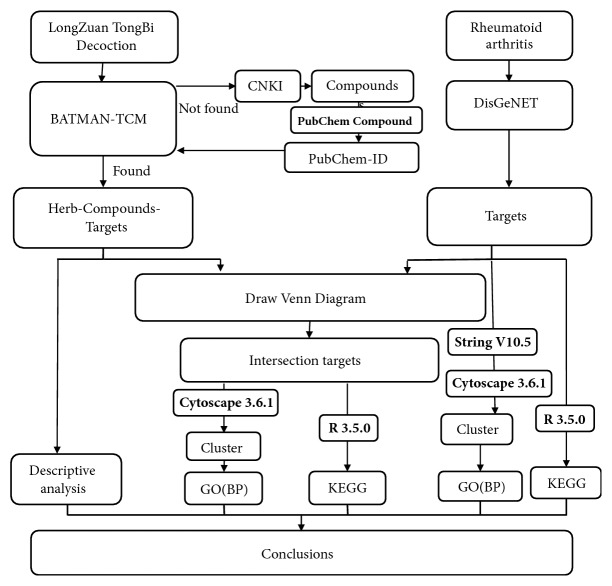
Technology roadmap.

In this study, the ClusterProfiler package of R3.5.0 [20] was adopted to conduct KEGG pathway enrichment analysis on overlapping target genes. Hypergeometric distribution was used for enrichment analysis, and Benjamini-Hochberg correction was selected for multiple testing correction of* p* value in enrichment analysis. Target analysis was based on the enrichment analysis of functional items to investigate the potential biological functions and involved biological pathways. The technology roadmap is described in Figure 1.

## 3. Results

### 3.1. Analysis of RA Target Network 

#### 3.1.1. RA Target Network

**Figure 2:**
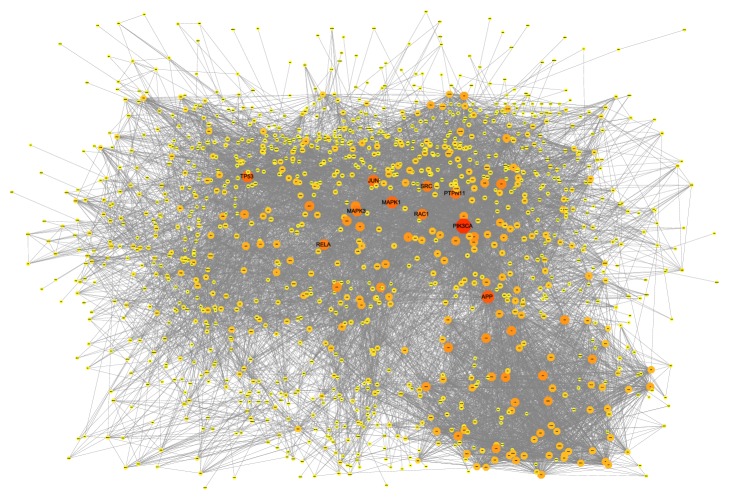
RA target PPI network.

There are 1,254 nodes and 11,181 edges in the RA target PPI network. The closer the nodes are to red and the larger the nodes are, the higher the degree of freedom they have. This demonstrates that these genes are closely related to other genes in the network, suggesting that these genes may play an important role in RA. Pathogenic factors may directly influence RA-related genes or indirectly influence RA-related genes by affecting these genes, thereby affecting the development of RA, which suggests that these genes may be the key or central genes. The top 10 proteins with the highest degree of freedom are PIK3CA, APP, MAPK1, TP53, JUN, PTPN11, RAC1, RELA, SRC, and MAPK3. The respective degrees of freedom are 164, 132, 122, 118, 115, 111, 104, 103, 103, and 103. The details are described in Figure 2.

#### 3.1.2. Clusters of RA Target Network

**Figure 3:**
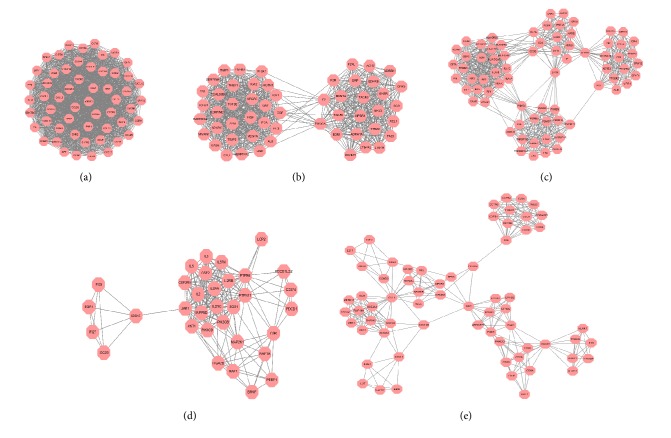
Clusters of RA target PPI network.

Five clusters were found after RA target network was analyzed through MCODE (K-Core=10). This demonstrates that these clusters may be the most relevant to RA in studies at present. The details are described in Table 1 and Figure 3.

#### 3.1.3. Enrichment Analysis of RA Target Network

The biological process (BP) enrichment analysis (p=0.05) of 5 clusters was conducted, and we found the following.

Cluster 1 contains 394 biological processes of which those associated with RA mainly include regulation of chemotaxis in immune cells, immune cell migration, angiogenesis, apoptosis, enzyme activity, and immune responses. The details are described in Table S02-1.

Cluster 2 contains 1,392 biological processes of which those associated with RA mainly include regulation of immune cell migration, chemotaxis in immune cells, cell secretion, biosynthesis of tumor necrosis factor (TNF), enzyme activity, hormone secretion, ossification, apoptosis signaling pathway, immune responses, and phagocytosis. The details are described in Table S02-2.

Cluster 3 contains 847 biological processes of which those associated with RA mainly include regulation of TNF-mediated signaling pathway, immune cell activation, immune responses, immune cell proliferation, and Wnt signaling pathways. The details are described in Table S02-3.

Cluster 4 contains 443 biological processes of which those associated with RA mainly include regulation of immune cell activation, inflammatory cell proliferation, and apoptosis signaling pathway. The details are described in Table S02-4.

**Figure 4:**
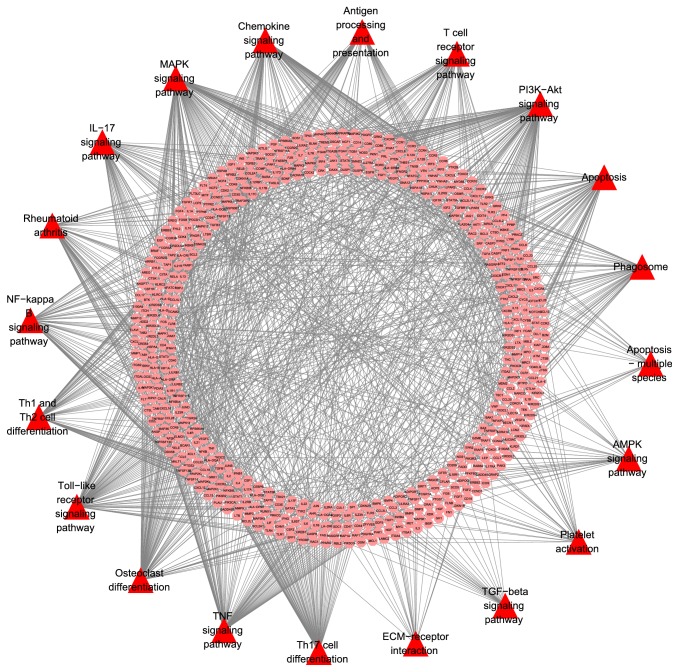
Signaling pathways in RA.

Cluster 5 contains 486 biological processes of which those associated with RA mainly include regulation of immune cell activation, inflammatory cell proliferation, immune responses, and apoptosis. The details are described in Table S02-5. The pathway analysis of all RA target genes (*p*=0.05) was carried out, and 24 pathways associated with RA were found. The details are described in Figure 4, and more information is described in Table S03.

In the aforementioned biological processes, regulation of immune responses, immune cell activation, and immune cell proliferation contributes to RA through joint damage caused by boosting immune responses and promoting inflammatory responses; regulation of inflammatory cell proliferation promotes RA through direct joint damage; dysregulation of apoptosis and phagocytosis promotes RA by giving rise to the dysplasia of angiogenesis and provides nutrition pathways for cell hyperplasia, which aggravates the development of RA; abnormal ossification of joints contributes significantly to the joint deformity of RA patients.

In the aforementioned pathways, Th17 cell differentiation, IL-17 signaling pathway, and Chemokine signaling pathway participate in the pathological process of RA through inflammatory response; TNF signaling pathway, NF-kappa B signaling pathway, MAPK signaling pathway, PI3K-Akt signaling pathway, apoptosis, apoptosis-multiple species, and phagosome participate in the pathological process of RA by influencing the apoptosis, proliferation, inflammatory response, and autophagy of synovial cells; osteoclast differentiation and AMPK signaling pathway participate in the pathological process of RA by leading to joint deformity through the damage of joint cartilage and bone; Toll-like receptor signaling pathway, Th1 and Th2 cell differentiation, T cell receptor signaling pathway, TGF-beta signaling pathway, and B cell receptor signaling pathway participate in the pathological process of RA by regulating innate and adaptive immunity and influencing the proliferation of synovioblast and pathological angiogenesis; Jak-STAT signaling pathway, FoxO signaling pathway, HIF-1 signaling pathway, and cAMP signaling pathway play an important role in the proliferation and apoptosis of synovial cells, osteocyte differentiation, and immunoregulation as they accept the activation of inflammatory factors and transmit signals to the corresponding targets; rheumatoid arthritis signaling pathway exhibits the pathological process of RA in many aspects, including abnormal activation of immune system, abnormal inflammatory response, abnormal proliferation of synovial cells, pannus formation, and osteoclast differentiation.

### 3.2. Analysis of LZTB Target Network

**Figure 5:**
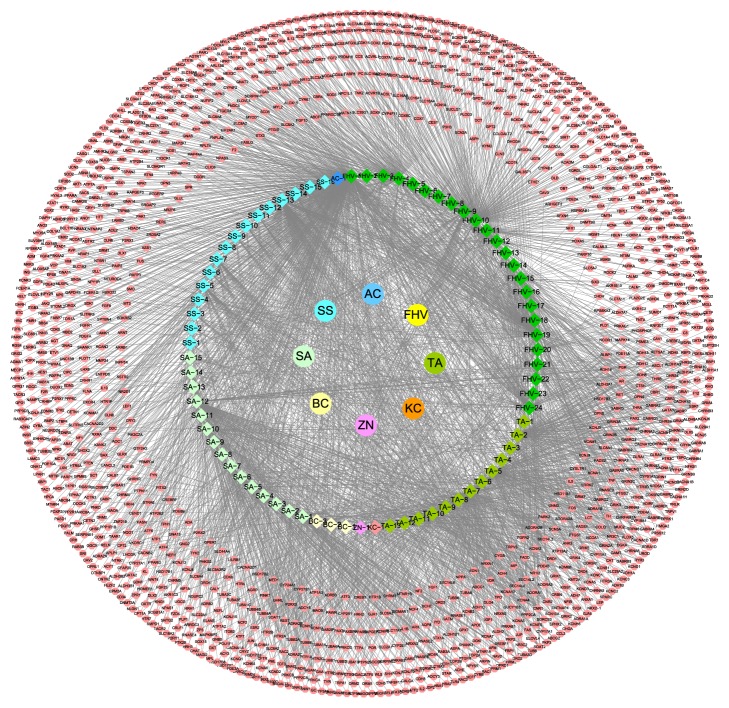
LZTB target network.

With cutoff set at 10,* p* value set at 0.05, 74 known compounds in LZTB and 2,987 target genes were retrieved. For* Toddalia asiatica* (TA), there are 13 compounds and 441 target genes: Chelidimerine (TA-1), Citronellol (TA-2), Isopinocamphone (TA-3), Mexolide (TA-4), Diosphenol (TA-5), Eugenol (TA-6), Alpha-Pinene (TA-7), Oxychelerythrine (TA-8), Tohogenol (TA-9), Robustine (TA-10), Skimmianine (TA-11), Citronellyl Acetate (TA-12), and Eugenol Methyl Ether (TA-13); for* Kadsura coccinea* (KC), there is 1 compound and 20 target genes: Schisantherin L (KC-1); for* Zanthoxylum nitidum* (ZN) there is 1 compound and 11 target genes: Oxynitidine (ZN-1); for* Bauhinia championii* (BC), there are 3 compounds and 24 target genes: Sinensetin (BC-1), 5,7,3′,4′,5′-Pentamethoxyflavone (BC-2), and 5,6,7,3′,4′,5′-Hexamethoxyflavone (BC-3); for* Sinomenium acutum* (SA), there are 15 compounds and 397 target genes: Stepholidine (SA-1), Magnoflorine (SA-2), Stepharine (SA-3), Dispegatrine (SA-4), Disinomenine (SA-5), Isosinomenine (SA-6), Michelalbine (SA-7), Magnograndiolide (SA-8), Michelenolide (SA-9), Sinactine (SA-10), Tuduranine (SA-11), Stigmasterol (SA-12), Sinomontanine D (SA-13), Gamma-sitosterol (SA-14), and Sinomenine (SA-15); for* Spatholobus suberectus* (SS), there are 16 compounds and 361 target genes: Licochalcone A (SS-1), Isoliquiritigenin (SS-2), Biochanin A (SS-3), Genistein (SS-4), Vestitol (SS-5), Afrormosin (SS-6), Isosativan (SS-7), Daidzein (SS-7), Formononetin (SS-8), Calycosin (SS-9), Prunetin (SS-10), Campesterol (SS-11), Stigmasterol (SS-12), Odoratin (SS-13), Pendulone (SS-14), and Medicagol (SS-15); for* Alangium chinense* (AC), there is 1 compound and 71 target genes: (+-)-Anabasine (AC-1); and for* Ficus hirta Vahl* (FHV), there are 24 compounds and 1,662 target genes: Palmitic acid (FHV-1), Meranzin hydrate (FHV-2), Quercetin (FHV-3), Oleic acid (FHV-4), Luteolin (FHV-5), Bergapten (FHV-6), Umbelliferone (FHV-7), Kaempferol (FHV-8), Sitosterol (FHV-9), Ethyl acetate (FHV-10), Methyleugenol (FHV-11), Narigenin (FHV-12), Hesperidin (FHV-13), p-hydroxybenzoic acid (FHV-14), Tricin (FHV-15), Acacetin (FHV-16), 1,2-benzenedicarboxylic acid, bis(2-methylpropyl) ester (FHV-17), *α*-amyrin acetate (FHV-18), Physcion (FHV-19), Cyclomorusin (FHV-20), Linoleic acid (FHV-21), *β*-amyrin acetate (FHV-22), Apigenin (FHV-23), and 4-hydroxy-3-methoxybenzoic acid (FHV-24). The details are described in Figure 5, and concrete data are described in Table S04.

The aforementioned results indicate that 8 herbs in LZTB and 67 compounds in the 8 herbs possibly are the material bases that play a key pharmacological role.

### 3.3. Analysis of Compound Target-RA Target Network

#### 3.3.1. Compound Target-RA Target Network

After the intersection process, we found there is an overlap between 8 herbs in LZTB and RA.

Specifically, 12 compounds in* Toddalia asiatica* (TA) have overlapping genes with RA: Citronellol (TA-2): SHBG, RET, ALDH1A2, ESR1, RHO, GATA3, ALDH1A1, CACNA1S, PGR, VCAM1, ALDH1A3, LRAT, AR; Isopinocamphone (TA-3): AR; Mexolide (TA-4): IL5, TNF, MUC2, RNASE3, NFKB1; Diosphenol (TA-5): ANXA1, ADORA2A, GRIK4, FADS2, NR3C1, CYP19A1, TH, TNF, PTGS2, ESR1, PARK2, TRPV1, TNFSF11, FOS, PGR, CYP17A1, NFKB1, HSD11B1, PTGS1, AR, PDE4A; Eugenol (TA-6): ALOX5, CNR1, CNR2, SEC14L2, NR1I2; Alpha-Pinene (TA-7): EDN1, IGF1, LEP, APOE, FAS, ALDH1A2, ASPA, MIP, ESR1, PPARD, SCGB1A1, FADD, KCNA3, ALDH1A1, PGR, CYP17A1, ALDH1A3, CAT, IL1B, SPARC, LRAT; Oxychelerythrine (TA-8): ADRA1A, PTGS2, ADRA2B, PTGS1; Tohogenol (TA-9): VDR, AR; Robustine (TA-10): ALOX5, FGF1, CHGA, PTPN2, CNR1, ABCB1, SOAT1, ABCG2, CNR2, SOCS1, SEC14L2, SERPINE1, NR1I2, TTPA, CCL3; Skimmianine (TA-11): MTNR1B, MTNR1A; Citronellyl Acetate (TA-12): ESR1, ADRA2B, PGR, AR; Eugenol Methyl Ether (TA-13): CNR1, CNR2.

One compound in* Kadsura coccinea* (KC) has 1 overlapping gene with RA: Schisantherin L (KC-1): TOP2B.

One compound in* Alangium chinense* (AC) has overlapping genes with RA: (+)-Anabasine (AC-1): CXCR4, PTK2B, HRH4, ADORA2A, ADRA1A, CYP19A1, TACR2, CHAT, ADRB2, LILRB1, ADRA2B, ADRB3, HTR2A.

One compound in* Zanthoxylum nitidum *(ZN) has overlapping genes with RA: Oxynitidine (ZN-1): ADRA1A, PTGS2, ADRA2B, PTGS1.

Three compounds in* Bauhinia championii *(BC) have overlapping genes with RA: Sinensetin (BC-1): IL5, TNF, MUC2, RNASE3, NFKB1; 5,7,3′,4′,5′-Pentamethoxyflavone (BC-2): IL5, TNF, MUC2, RNASE3, NFKB; 5,6,7,3′,4′,5′-Hexamethoxyflavone (BC-3): IL5, TNF, MUC2, RNASE3, NFKB1.

Fourteen compounds in* Spatholobus suberectus *(SS) have overlapping genes with RA: Licochalcone A (SS-1): HSD17B1, CNR1, PDE3A, SOAT1, CNR2; Isoliquiritigenin (SS-2): HSD17B1, ALOX5, PPARG, PTGS2, ESR1, CFTR, PTGS1; Biochanin A (CS-3): ALOX5, CNR1, SOAT1, CNR2, SEC14L2, NR1I2; Genistein (SS-4): HSD17B1, ESR1, CACNA1S, PGR, NR1I2, AR, ESR2; Vestitol (SS-5): ALOX5, FGF1, CHGA, PTPN2, CNR1, ABCB1, SOAT1, ABCG2, CNR2, SOCS1, SEC14L2, SERPINE1, NR1I2, TTPA, CCL3; Afrormosin (SS-6): ALOX5, CNR1, SOAT1, CNR2, SEC14L2, NR1I2; Isosativan (SS-7): ALOX5, FGF1, CHGA, PTPN2, CNR1, ABCB1, SOAT1, ABCG2, CNR2, SOCS1, SEC14L2, SERPINE1, NR1I2, TTPA, CCL3; Formononetin (SS-9): ALOX5, CNR1, SOAT1, CNR2, SEC14L2, NR1I2; Calycosin (SS-10): ALOX5, CNR1, SOAT1, CNR2, SEC14L2, NR1I2; Prunetin (SS-11):ALOX5, CNR1, SOAT1, CNR2, SEC14L2, NR1I2; Campesterol (SS-12): ANXA1, NR3C1, ESR1, VDR, PGR, AR; Stigmasterol (SS-13): EDN1, IGF1, LEP, APOE, FAS, ALDH1A2, ASPA, MIP, ESR1, PPARD, SCGB1A1, FADD, KCNA3, ALDH1A1, PGR, CYP17A1, ALDH1A3, CAT, IL1B, SPARC, LRAT; Odoratin (SS-14): ALOX5, CNR1, SOAT1, CNR2, SEC14L2, NR1I2; Pendulone (SS-15): ADRA1A, CNR1, SOAT1, CNR2, ADRA2B.

Fourteen compounds in* Sinomenium acutum* (SA) have overlapping genes with RA: Stepholidine (SA-1): NCF4, RAC1, RAC2, NCF2, NCF1, ESR1, ADRA2B, CYBB, HTR2A, ESR2; Magnoflorine (SA-2): ALOX5, ADRA2B, SEC14L2, HTR2A, NR1I2; Dispegatrine (SA-4): ADRA2B, HTR2A; Disinomenine (SA-5): SHH, ESR1, CREB1, TLR4, HTR2A, ESR2; Isosinomenine (SA-6): SHH, ESR1, CREB1, TLR4, HTR2A, ESR2; Michelalbine (SA-7): NFATC1, NPPB, ADORA2A, ADRA1A, SELE, TNF, ADRB2, VEGFA, ADRA2B, IL2, FOS, ATF2, HIF1A, VCAM1, ADRB3, HTR2A, JUN, HBA1; Magnograndiolide (SA-8): ATP1A1, ITGB2, HMGCR, AR, ITGAL; Michelenolide (SA-9): ADRA1A; Sinactine (SA-10): NCF4, RAC1, ELANE, RAC2, HRH4, NCF2, ADRA1A, SLC22A1, NCF1, ADRA2B, CYBB, TLR4, HTR2A; Tuduranine (SA-11): ADRA2B, HTR2A; Stigmasterol (SA-12): EDN1, IGF1, LEP, APOE, FAS, ALDH1A2, ASPA, MIP, ESR1, PPARD, SCGB1A1, FADD, KCNA3, ALDH1A1, PGR, CYP17A1, ALDH1A3, CAT, IL1B, SPARC, LRAT; Sinomontanine D (SA-13): UGCG, GAA, PADI4; Gamma-Sitosterol (SA-14): CALB1, KL, NR3C1, GC, ESR1, VDR, PML, PGR, CYP17A1, NFKB1, AR, SNAI2, BAX; Sinomenine (SA-15): ESR1, ESR2.

**Figure 6:**
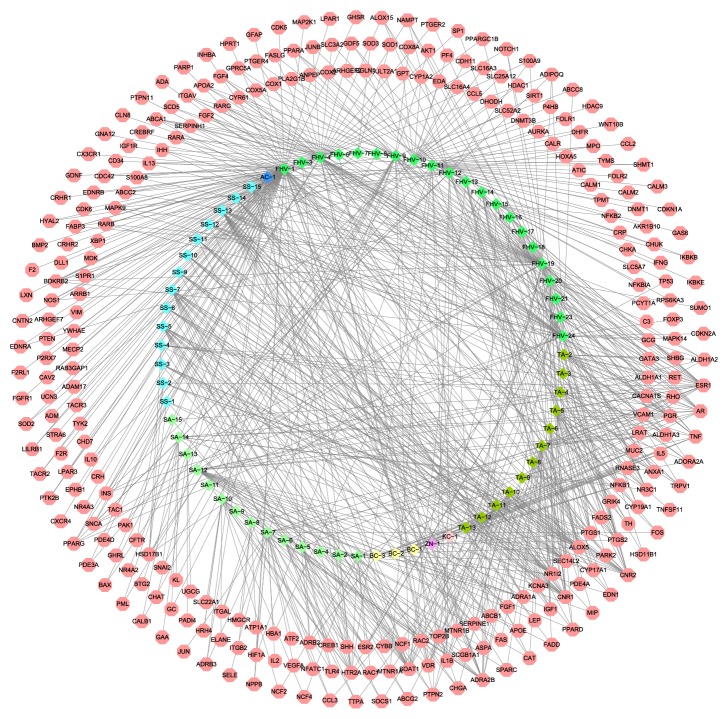
Overlapping target genes between LZTB and RA.

Twenty-one compounds in* Ficus hirta Vahl* (FHV) have overlapping genes with RA: palmitic acid (FHV-1): BTG2, EDN1, IGF1, PAK1, SOD2, FGFR1, MTNR1B, NR4A2, F2RL1, EDNRA, GHRL, CNTN2, ADRA1A, LXN, TACR2, F2, PTGS2, BMP2, TAC1, PDE4D, SNCA, NR4A3, PARK2, HYAL2, EPHB1, TRPV1, INS, CRHR1, LPAR3, GDNF, CRH, IL10, CHD7, TYK2, F2R, STRA6, TACR3, ALDH1A3, CAT, ADAM17, ADRB3, ADM, UCN3, CAV2, IL1B, HTR2A, CX3CR1, P2RX7, GNA12, PTEN, PTK2B, RAB3GAP1, CLN8, MECP2, ARHGEF7, NOS1, PTPN11, NPPB, YWHAE, BDKRB2, ADORA2A, CHGA, DLL1, CRHR2, CNR1, FABP3, CDK6, TH, VIM, EDNRB, CDC42, ARRB1, ADRB2, ADA, PARP1, CD34, INHBA, LILRB1, ADRA2B, S1PR1, FOS, MDK, IGF1R, HPRT1, HIF1A, XBP1, GFAP, CREBRF, CDK5, MAP2K1, PTGS1, LPAR1, GHSR; quercetin (FHV-3)ADRA2B, HTR2A; oleic acid (FHV-4): IGF1, ANXA1, NOS1, SHBG, ABCA1, BDKRB2, SCD5, PPARG, ALOX15, RARB, RET, FADS2, ALDH1A2, MAPK9, PTPN2, NR3C1, FABP3, TNF, ABCC2, ITGAV, APOA2, PTGS2, FGF4, GPRC5A, ESR1, SNCA, S100A8, IL13, PTGER4, TRPV1, VDR, PPARD, IHH, FASLG, INS, RARA, SERPINH1, PPARA, GATA3, NAMPT, PTGER2, SP1, JUNB, FGF2, ALDH1A1, SLC3A2, GDF5, PGR, RARG, ALDH1A3, IL1B, PTGS1, PPARGC1B, AR, NOTCH1, CYR61, S100A9, ADIPOQ; bergapten (FHV-6): RARB, TNF, VDR, RARA, RARG; umbelliferone (FHV-7): SOD2, SOD3, SOD1; kaempferol (FHV-8): HSD17B1; sitosterol (FHV-9): COX5A, CALB1, ABCA1, KL, COX8A, ABCB1, NR3C1, GC, ABCC2, COX1, ESR1, SOAT1, VDR, HMGCR, PML, PLA2G1B, ANPEP, PGR, CYP17A1, COX2, AKT1, NFKB1, ABCC8, AR, SNAI2, BAX, ITGAL; ethyl acetate (FHV-10): HSD17B1, PF4, BDKRB2, ARHGEF2, HDAC9, TH, EGLN1, SULT2A1, CDC42, CDH11, WNT10B, GPT, CCL2, SHMT1, CYP1A2, IL10, EDA, SLC16A4, SLC16A3, CCL5, SLC25A12, HDAC1, SIRT1, P4HB, DHODH, SLC52A2; methyleugenol (FHV-11): MTNR1B, DNMT3B, FOLR1, AURKA, DHFR, ABCC2, MPO, CALR, HOXA5, ESR1, TYMS, PDE3A, FOLR2, CALM2, MTNR1A, SHMT1, HYAL2, DNMT1, HIF1A, CALM3, CDKN1A, IL1B, HTR2A, GAS6, ATIC, CALM1, PDE4A; p-hydroxybenzoic acid (FHV-12): ANXA1, ALOX5, AKR1B10, ABCA1, BDKRB2, PPARG, ALOX15, MAPK9, PTPN2, TPMT, FABP3, TNF, MPO, ITGAV, CHUK, APOA2, PTGS2, SNCA, S100A8, IL13, INS, PPARA, NAMPT, PLA2G1B, IKBKB, IFNG, IKBKE, SERPINE1, IL1B, PTGS1, S100A9, ADIPOQ; tricin (FHV-13): ALOX5, CNR1, SOAT1, CNR2, SEC14L2, NR1I2; acacetin (FHV-14): ALOX5, ADORA2A, CNR1, NR3C1, ESR1, SOAT1, CNR2, SEC14L2, NR1I2, HBA1; 1,2-benzenedicarboxylic acid, bis(2-methylpropyl) ester (FHV-15): NFKB2, EDNRA, CRP, PTGS2, CHKA, SLC5A7, ADRA2B, IKBKB, TP53, NFKB1, NFKBIA, PTGS1, RPS6KA3; *α*-amyrin acetate (FHV-16): NR3C1, CYP19A1, ESR1, ADRA2B, PGR, AR; physcion (FHV-17): CNR1, PTGS2, ESR1, SOAT1, CNR2, PTGS1; cyclomorusin (FHV-18): CNR1, CNR2; linoleic acid (FHV-19): IGF1, ANXA1, NOS1, PCYT1A, SHBG, ABCA1, BDKRB2, SCD5, PPARG, ALOX15, RARB, RET, FADS2, ALDH1A2, MAPK9, PTPN2, NR3C1, FABP3, TNF, ABCC2, ITGAV, APOA2, PTGS2, FGF4, GPRC5A, SNCA, S100A8, IL13, PTGER4, TRPV1, VDR, PPARD, IHH, FASLG, INS, RARA, SERPINH1, PPARA, GATA3, NAMPT, PTGER2, SP1, JUNB, FGF2, ALDH1A1, SLC3A2, GDF5, RARG, ALDH1A3, IL1B, HSD11B1, PTGS1, PPARGC1B, AR, NOTCH1, CYR61, S100A9, ADIPOQ; narigenin (FHV-20): HSD17B1, SUMO1, ALOX5, C3, CNR1, ESR1, SOAT1, CNR2, SEC14L2, AKT1, NR1I2, ESR2; *β*-amyrin acetate (FHV-21): NR3C1, CYP19A1, ESR1, ADRA2B, PGR, AR; hesperidin (FHV-23): ESR1, ADRB2, SOAT1, VDR, HTR2A, NR1I2, ESR2; 4-hydroxy-3-methoxybenzoic acid (FHV-24): ANXA1, FOXP3, ABCA1, BDKRB2, PPARG, ALOX15, RARB, MAPK9, MAPK14, TNF, ITGAV, PTGS2, FGF4, SNCA, VDR, IHH, INS, RARA, SERPINH1, PPARA, NAMPT, FGF2, GDF5, RARG, TP53, GCG, PTGS1, CDKN2A, ABCC8, NOTCH1, CYR61, ADIPOQ. The details are described in Figure 6 and Table S05.

The aforementioned results suggest that LZTB has intervention effects on RA as the 8 herbs in LZTB can have effects on RA through multiple compounds and targets.

#### 3.3.2. Clustering Analysis of LZTB Target-RA Target Network

**Figure 7:**
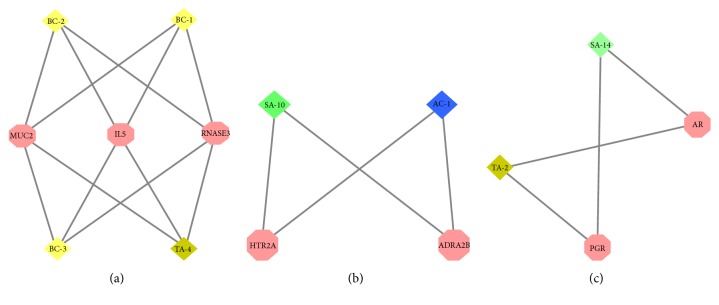
LZTB target-RA target network clusters.

We obtained 3 clusters after conducting clustering analysis for LZTB target-RA target network (K-core=2). The details are described in Figure 7 and Table S06.

Cluster 1 contains 4 compounds: BC-1, BC-2, BC-3, and TA-4; cluster 2 contains 2 compounds: A-10 and AC-1; cluster 3 contains 2 compounds: SA-14 and TA-2. Three clusters contain 8 compounds and 4 Zhuang herbs:* Toddalia asiatica* (TA),* Bauhinia championii* (BC),* Sinomenium acutum* (SA), and* Alangium chinense* (AC). It indicates that the 8 compounds in the 4 aforementioned Zhuang herbs play an important role in the treatment of RA.

Cluster 1 contains 3 genes: MUC2, IL5, and RNASE3; cluster 2 contains 2 genes: HTR2A and ARA2B; and cluster 3 contains 2 genes: AR and PGR. It indicates that the 7 aforementioned genes are key to the treatment of RA with LZTB.

#### 3.3.3. Enrichment Analysis of LZTB Target-RA Target Network

**Figure 8:**
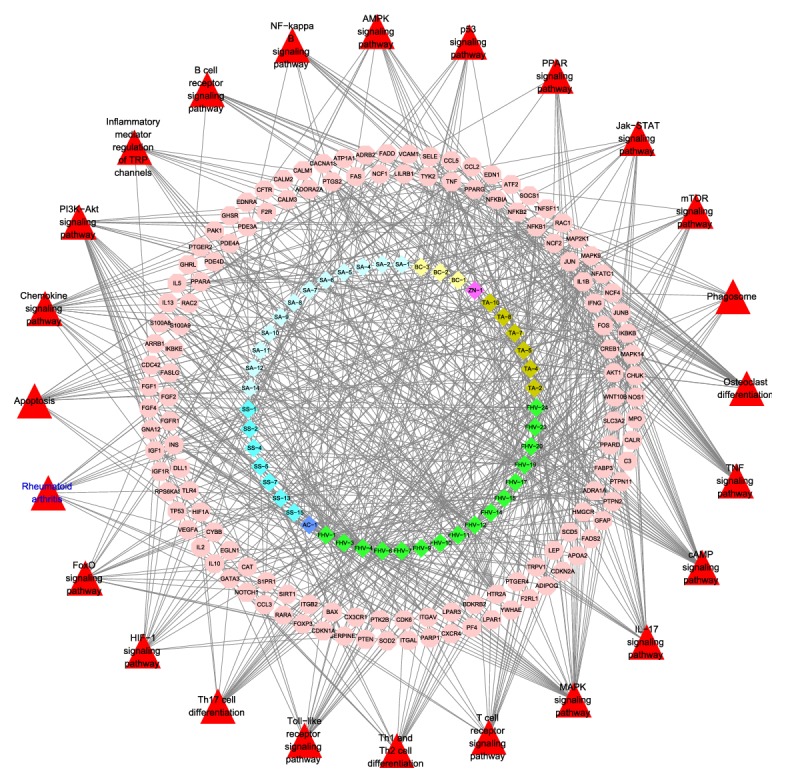
Pathways of LZTB target-RA target network.

The GO enrichment analysis of the aforementioned clusters (*p*=0.05) showed that cluster 1 contains 20 biological processes of which those associated with RA mainly include regulation of immunoglobulin secretion, immune responses, and B cell proliferation. The details are described in Table S07-1; cluster 2 contains 276 biological processes of which those associated with RA mainly include regulation of enzyme activity and metabolism. The details are described in Table S07-2; cluster 3 contains 142 biological processes of which those associated with RA mainly include regulation of hormone-mediated signaling pathways and apoptosis signaling pathways. The details are described in Table S07-3; after pathway analysis of aforementioned overlapping genes was conducted (*p*=0.05), 24 pathways associated with RA were found, and 7 herbs in LZTB participate in the regulations of pathways. The details are described in Figure 8 and Table S08.

**Figure 9:**
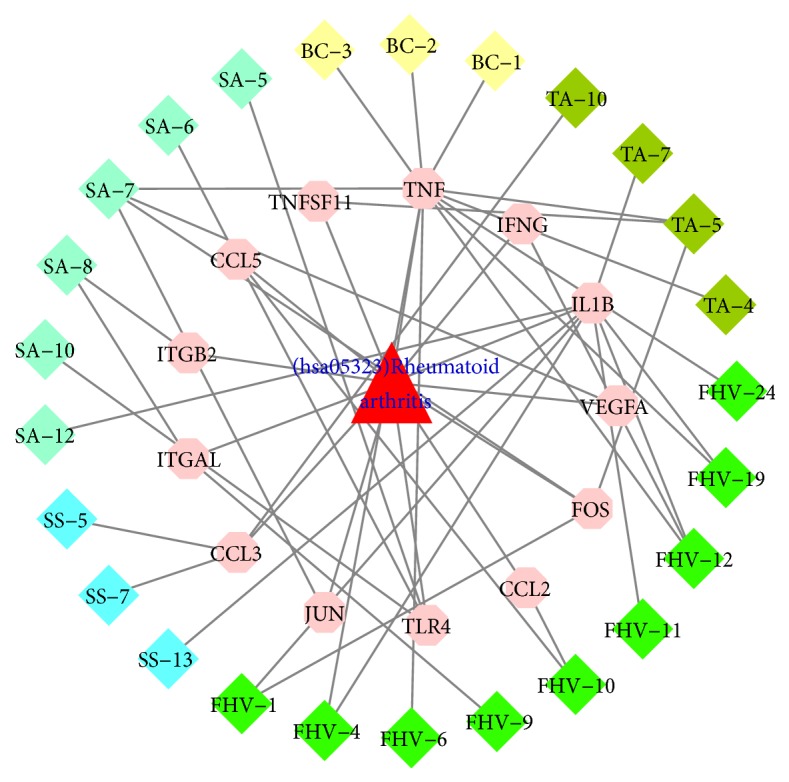
Rheumatoid arthritis pathway.

Among them, analysis of hsa05323 (rheumatoid arthritis) indicated that it is directly associated with RA. TA-4, TA-5, TA-7, TA-10, BC-1, BC-2, BC-3, SA-5, SA-6, SA-7, SA-8, SA-10, SA-12, SS-5, SS-7, SS-13,FHV-1, FHV-4, FHV-6, FHV-9, FHV-10, FHV-11, FHV-12, FHV-19, and FHV-24 are effective compounds in LZTB that directly intervene with RA pathways, and 13 genes are involved: CCL2, CCL3, CCL5, FOS, IFNG, IL1B, ITGAL, ITGB2, JUN, TLR4, TNF, TNFSF11, and VEGFA. The details are described in Figure 9 and Table S09. Compared with pathways of RA network, 3 different signaling pathways were found through pathway analysis of the network: inflammatory mediator regulation of TRP channels, PPAR signaling pathway, and mTOR signaling pathway.

TRP channels (transient receptor potential channels) are a group of nonselective cation channels throughout the body, and they are Ca 2 + permeable. TRP channels consist of more than 30 members that are divided into 7 subfamilies: TRPC, TRPV, TRPM, TRPA, TRPP, TRPML, and TRPN [21]. According to research, TRP channels have become drug targets for the treatment of RA as there are multiple TRP channels in rheumatoid arthritis synovial fibroblasts [22–26], including TRPV1, TRPA1, TRPC5, TRPM3, TRPM7, and TRPM8. There are many studies on TRPV1 and TRPA1. In the pathological process of RA, TRPV1 and TRPA1 mainly participate in the pain caused by inflammatory mediators and apoptosis of synovial cells [25, 27, 28]. TRPC5 mainly participates in the endogenous anti-inflammatory process of RA [29, 30]. As a sensor for steroid hormones, TRPM3 can inhibit the secretion of anti-inflammatory mediator-HA from synovial cells when stimulated by steroid hormone progesterone applied outside cells [31, 32]. TRPM7 participates in the pathological process of RA through the antagonism against neutrophils and synovial cells [33, 34]. TRPM8 plays an important role in the menthol-induced apoptosis of synovial cells [35]. In summary, current studies suggest that activation of the aforementioned channels can improve RA symptoms and prevent RA.

PPARs (peroxisome proliferation-activated receptors) are ligand-activated transcription factors, comprising of the following three subtypes: PPAR-*α*, PPAR-*β*, and PPAR-*γ*. PPAR-*γ* is more closely related to RA. According to research, the expression of PPAR-*γ* can be detected in synovial cells involved in rheumatoid arthritis. PPAR-*γ* agonists can inhibit the hyperplasia of synovial cells and induce their apoptosis [36, 37]. In addition, PPAR-*γ* agonists can inhibit the generation of key mediators in RA from macrophages, including IL-1*β*, IL-6, and TNF-*α* [36]. In conclusion, PPAR signaling pathway plays a role in treating RA by intervening with the pathological process of RA through the corresponding receptor agonists.

Serine/threonine-protein kinase mTOR (mammalian target of rapamycin) belongs to the PIKK (phosphoinostitide-3-kinase-related kinase) family, and it plays a key role in regulating cell growth, proliferation and survival. In RA-related mTOR signaling pathways, PI3K/Akt/mTOR signaling pathway is actively studied [38]. In the course of RA, platelet microparticles accumulate, and the activated products (e.g., PDGFR*α*) are released into articular cavity. Then, the activated PI3K in synovioblasts transmits signal to Akt. Regulating multiple transcription factors, the activated Akt helps with cell survival by inhibiting the expression of apoptosis gene (e.g., Fas-l) and the activity of proapoptotic protein (Bad) and enhancing the expression of antiapoptotic gene (e.g., NF—*κ*B) [39]. Akt activates mTOR via direct or indirect phosphorylation. The activated mTOR can upregulate cyclins to accelerate cell cycle and also regulate cell growth by inhibiting autophagy [40]. In summary, PI3K/Akt/mTOR signaling pathway participates in the pathological process of RA by inhibiting the apoptosis of synovioblasts, accelerating synovioblast cycle, and controlling the autophagy of synovioblasts. It can improve or control RA symptoms by downregulating this signaling pathway.

In conclusion, the three aforementioned signaling pathways of LZTB possibly act on RA.

## 4. Conclusion

Currently, conventional synthetic disease-modifying antirheumatic drugs remain the first choice for clinical treatment in RA. We found in this study that some compounds in LZTB are directly involved in the regulation of RA pathways and that they possibly are major compounds in LZTB for the treatment of RA, for example, Mexolide, Diosphenol, Alpha-Pinene, Robustine, Sinensetin, 5,7,3′,4′,5′-Pentamethoxyflavone, 5,6,7,3′,4′,5′-Hexamethoxyflavone, Stepholidine, Magnoflorine, Dispegatrine, Disinomenine, Isosinomenine, Michelalbine, Magnograndiolide, Michelenolide, Sinactine, Tuduranine, Stigmasterol, Vestitol, Daidzein, Odoratin, Palmitic acid, Oleic acid, Bergapten, Sitosterol, Ethylacetate, Methyleugenol, Narigenin, Physcion, and 4-hydroxy-3-methoxybenzoicacid. In this study, we applied network-based computational methods to predict and expound the molecular synergy of LZTB for RA. It will provide new ideas for further research on ethnopharmacology, Chinese medicinal herbs and ethnic compounds. The targets, clusters, biological processes, and pathways associated with RA were discovered through this study. LZTB target-RA target network exhibited the effective chemical compounds, potential pharmacology, and molecular mechanism of LZTB for treating RA and also justified the composition of LZTB.

## Figures and Tables

**Table 1:** Clusters of RA target PPI network.

Cluster	Score	Nodes	Edges	Gene IDs
1	55	55	1485	ADRA2B, APP, POMC, CXCL13, CXCL2, CXCR5, S1PR3, CCR1, S1PR1, LPAR1, C5, C5AR1, CXCR7, CCR3, C3, MTNR1B, CCR6, CCL5, MTNR1A, CCL20, FPR2, PPBP, CCR2, BDKRB2, ANXA1, CCR5, SSTR4, HRH4, CCL21, SST, PDYN, SAA1, CCR10, CCL28, LPAR2, CXCL12, PF4, CCR4, LPAR3, CXCR1, S1PR2, CXCL10, CXCR3, CXCL11, CXCL9, CXCL6, CCR9, CCL25, CXCR4, CXCL16, CXCR6, CNR1, CXCR2, CXCL1, CNR2
2	27.283	54	723	IGF2, HGF, ALB, TACR2, FIGF, VEGFC, TGFB3, CLU, SERPINF2, GRK5, ADRA1A, A2M, SERPINA1, EDNRA, F2, LTB4R, HTR2A, VWF, VEGFA, TAC1, GHSR, TIMP3, FGB, FN1, TGFB2, GCG, XCL1, TIMP1, GAS6, RARRES2, PIK3R3, PIK3CA, MMRN1, SPARC, F2RL1, EGF, LGALS3BP, ADRBK1, ACTN1, NPSR1, THBS1, TGFB1, F2R, SERPINE1, PLG, GRP, BRS3, TACR3, TACR1, LTB4R2, EDN1, F13A1, EDNRB, IGF1
3	19.268	72	684	CRHR1, IRF8, IRF1, LTBR, LTB, CGA, LTA, WNT5A, HLA-DRA, PTH, PTHLH, IRF4, CRHR2, ADRB3, ADORA2A, CD4, ADM, IRF3, TNFRSF11A, TNFSF11, PSMD9, HLA-E, PTGER2, CFTR, TF, VCAM1, TNFRSF1B, PTGER4, GBP6, CLTA, CD44, VIPR1, VIP, TNFRSF13C, TNFSF13B, FCGR1A, PSMB5, APOB, PSMD7, IRF5, HLA-DQA2, HLA-DPB1, TNFSF14, HLA-C, HLA-B, B2M, RAMP2, CALCRL, ADRB2, TNF, TRIM21, NCAM1, AGFG1, ARRB1, SYT1, EGFR, CRH, GPR15, LRP2, PSMB9, PSMB8, CIITA, ICAM1, HLA-DRB1, CD40LG, HLA-DRB5, PML, HLA-A, CD3G, HSPA8, LDLR, PSMD12
4	12.312	33	197	IL2RB, IFI27, PDCD1, IL3, AKT1, PDCD1LG2, CSF2, CSF2RA, IL5, IFI35, PTPN11, ISG20, CSK, BRAF, JAK1, ITGA2B, RAF1, RAP1A, PTPN6, ISG15, EGR1, IL2RA, SOS1, PIK3CD, PIK3CB, IL2RG, INPP5D, LCP2, PEBP1, CD274, IL5RA, MAP2K1, IL2
5	8.406	65	269	STAT1, KLRC2, EPHB1, UBA1, UBE2L3, COPB1, COPB2, CD59, SOCS3, SRC, CUL1, ZBTB16, CD8A, ARHGEF7, IL6ST, BTK, CCND3, PARK2, INS, E2F1, KLRK1, IL27RA, CD28, FOLR1, DCTN6, DCTN5, STAT5B, NFKBIB, PAK1, PRKCQ, REL, EPHB2, ZNF645, UBA7, CD55, NFKB2, TP53, SOCS1, NFKB1, NFKBIA, CD86, RELB, TYROBP, TMED7, TREM2, KLRD1, EFNB2, E2F2, RNF19A, EFNB1, GORASP1, USO1, PAK3, ITCH, NFKBIE, PRF1, CDK4, COG6, GRAP2, CD80, CD3E, PRKCZ, CDK6, EBI3, IL27

## Data Availability

The data used to support the findings of this study are included within the Supplementary Materials.

## Supplementary Materials

Supplementary 1
** Table S01**:** The detailed information about compounds acquired from CNKI.**

Supplementary 2
** Table S02-1**: Cluster 1 data of biological process (BP) enrichment analysis of RA target network.** Table S02-2**: Cluster 2 data of biological process (BP) enrichment analysis of RA target network.** Table S02-3**: Cluster 3 data of biological process (BP) enrichment analysis of RA target network.** Table S02-4**: Cluster 4 data of biological process (BP) enrichment analysis of RA target network.** Table S02-5**: Cluster 5 data of biological process (BP) enrichment analysis of RA target network.

Supplementary 3
** Table S03**: The pathway analysis data of all RA target genes.

Supplementary 4
** Table S04**: The analysis data of LZTB target network.

Supplementary 5
** Table S05**: Data of compound target - RA target network.

Supplementary 6
** Table S06**: Cluster data of LZTB target-RA target network.

Supplementary 7
** Table S07-1**: Cluster 1 data of gene ontology (GO) enrichment analysis of LZTB target-RA target network.** Table S07-2**: Cluster 2 data of gene ontology (GO) enrichment analysis of LZTB target-RA target network.** Table S07-3**: Cluster 3 data of gene ontology (GO) enrichment analysis of LZTB target-RA target network.

Supplementary 8
** Table S08**: The pathway analysis data of LZTB target-RA target network.

Supplementary 9
** Table S09**: Data of hsa05323 (rheumatoid arthritis).
